# Preparation, Mechanical and Thermal Properties of Cement Board with Expanded Perlite Based Composite Phase Change Material for Improving Buildings Thermal Behavior

**DOI:** 10.3390/ma8115408

**Published:** 2015-11-13

**Authors:** Rongda Ye, Xiaoming Fang, Zhengguo Zhang, Xuenong Gao

**Affiliations:** Key Laboratory of Enhanced Heat Transfer and Energy Conservation, the Ministry of Education, School of Chemistry and Chemical Engineering, South China University of Technology, Guangzhou 510640, China; ye.rongda@mail.scut.edu.cn (R.Y.); cexmfang@scut.edu.cn (X.F.); cexngao@scut.edu.cn (X.G.)

**Keywords:** expanded perlite, composite phase change material, building energy conservation

## Abstract

Here we demonstrate the mechanical properties, thermal conductivity, and thermal energy storage performance of construction elements made of cement and form-stable PCM-Rubitherm^®^ RT 28 HC (RT28)/expanded perlite (EP) composite phase change materials (PCMs). The composite PCMs were prepared by adsorbing RT28 into the pores of EP, in which the mass fraction of RT28 should be limited to be no more than 40 wt %. The adsorbed RT28 is observed to be uniformly confined into the pores of EP. The phase change temperatures of the RT28/EP composite PCMs are very close to that of the pure RT28. The apparent density and compression strength of the composite cubes increase linearly with the mass fraction of RT28. Compared with the thermal conductivity of the boards composed of cement and EP, the thermal conductivities of the composite boards containing RT28 increase by 15%–35% with the mass fraction increasing of RT28. The cubic test rooms that consist of six boards were built to evaluate the thermal energy storage performance, it is found that the maximum temperature different between the outside surface of the top board with the indoor temperature using the composite boards is 13.3 °C higher than that of the boards containing no RT28. The thermal mass increase of the built environment due to the application of composite boards can contribute to improving the indoor thermal comfort and reducing the energy consumption in the buildings.

## 1. Introduction

Buildings are one of the leading sectors in the energy consumption in the developed countries. Taking the European Union as an example, the buildings sector consumes around 40% of the total fossil energy and produces nearly 40% of the total CO_2_ emissions. Most of it is due to the increase in the living standard and in occupants’ comfort demands, mainly for heating and cooling [1]. Improving the energy efficiency of buildings has a significant benefit for energy-saving and emission-reduction on the earth. In particular, nowadays the trend in the commercial buildings is to decrease the wall thickness to reduce the materials consumed, the transport costs, and the construction time. The main disadvantage of these lightweight buildings is the low thermal mass, resulting in large temperature fluctuations indoors. A phase change material (PCM) can absorb or release a large quantity of latent heat when it changes phase from solid state to liquid state or vice versa, and has been commonly used in thermal energy storage systems [2,3,4,5]. The integration of a PCM in building ceilings, walls, and floors to store significant amounts of thermal energy can compensate for the small storage capacity of the lightweight buildings and thus decrease the frequency of internal air temperature swings, leading to an improvement in the human comfort and a reduction in the energy consumption for the buildings.

Since 1982, the incorporation of PCMs into building fabrics has been investigated as a potential technology for minimizing the energy consumptions in buildings [6,7,8]. The selection of PCMs is almost directed towards the use of organic materials in an effort to avoid some of the problems inherent in inorganic materials, such as the need for special container due to their corrosivity, tendency of super cooling, segregation, *etc.* Up to now, three general methods have been proposed to integrate organic PCMs into construction elements [9,10,11], which are the direction immersion of conventional wallboards into molten PCMs [12,13,14,15,16], the integration of microencapsulated PCMs with ordinary building materials [17,18,19,20,21], and the combination of building materials with a shape-stabilized PCM that is usually prepared by blending an organic PCM with a supporting material [22,23,24]. Although the first one is simple and low cost, the impregnated wallboards are inflammable owing to the diffusion of the liquid PCMs to the surfaces of the wallboards, especially after the PCMs experienced several heating-cooling cycles. The second one suffers from the complicated polymerization processes and the high costs related to the microencapsulation of PCMs. Compared with the two mentioned methods, the third one has the advantages of simple process, low cost, and a large variety of supporting matrices available, such as high density polyethylene, expanded graphite, bentonite, and so on [25,26,27,28,29,30,31,32].

Expanded Perlite (EP), a light-weight (unit volume weight: 0.05–0.30 g/mL), odorless, and heat expanded volcanic mineral, has been commonly used as the ultra-light-weight building material to improve the structure of buildings because of its excellent heat insulation capacity (thermal conductivity rate: 0.03–0.05 kcal/mh °C) [33], environmentally safety, and abundant availability. The properties of high porosity, large surface area, low sound transmission, excellent fire-resistance, and low moisture retention make EP to be a good and cheap supporting matrix for preparing form-stable composite PCMs. Karaipekli and Sari groups [34,35,36] prepared several kinds of EP-based composite PCMs including the capric–myristic acid (CA-MA)/EP, CA/EP, lauric acid (LA)/EP, paraffin/EP and fatty acid esters/EP composites, the thermal properties, thermal reliability and the thermal conductivity of the composite PCMs are determined. The maximum PCMs absorptions of EP are obtained without melted PCMs seepage from the composites, and therefore these mixtures are described as the form-stable composites. Jiao *et al.* [37] have prepared the binary eutectic of LA-stearic acid (SA)/EP composite PCMs by vacuum impregnation. The structure and properties of the composite PCMs are characterized. The results show that the binary eutectic of fatty acids has been composed with the porous skeleton EP completely in a physical method. Chung *et al.* [38] prepared and characterized thermal properties and thermal reliability of form-stable composite PCMs composed of *n*-octadecane, expanded vermiculite, and perlite for thermal energy storage. The results showed that the prepared composite PCMs showed good compatibility between *n*-octadecane and the expanded vermiculite and pearlite, the thermal conductivities of composites were reduced, the large latent heat capacity and original phase change temperatures were maintained. Zhang *et al.* [39] prepared LA-palmitic acid (PA)-SA/EP composite PCMs by vacuum impregnation method. The maximum mass ratio of LA-PA-SA retained in EP was found as 55 wt %. They found that the thermal conductivity of LA-PA-SA/EP was increased by 95% by adding 2 wt % expanded graphite, and the thermal energy storage/release rates were also increased. Sun *et al.* [40] prepared the form-stable PCMs by absorbing paraffin into EP method. Graphite as additive was added into the form-stable PCMs to improve thermal conductivity. The results showed that the thermal conductivity of the form-stable PCMs was increased as much as 192% by graphite with mass fraction of 5%. Ramakrishnan *et al.* [41] have developed a novel thermal energy storage composite by impregnating paraffin into hydrophobic coated expanded perlite (EPO) granules. They found that no paraffin leakage was observed for novel paraffin/EPO containing 50% by weight of paraffin in the composite. Microstructural and mechanical properties were studied for the compatibility of hydrophobic coated PCM composite in concrete. Peng *et al.* [42] prepared form-stable composite PCMs for use in wallboards absorbing SA and LA eutectic mixtures into the pores of EP. The microstructure, thermal properties and the thermal reliability of the composite PCMs were characterized. Their results indicated that the maximum SA-LA absorption of the EP was as high as 65 wt % without any melted SA-LA leakage. A gypsum-based building wallboard containing 6 wt % SA-LA/EP had a low density (0.924 g/cm^3^), high mechanical strength (2.19 MPa), and remarkable heating preservation performance. Zhang *et al.* [43] prepared the CA-PA/EP composite PCM and fabricated the thermal-regulated gypsum boards by adding the prepared composite PCM. They found that the higher the composite PCM volume content, the smaller the thermal conductivity of the gypsum board. The bending strength and compressive strength reduced gradually with an increase of the volume fraction of the composite PCM. He *et al.* [44] prepared the form/stable CA-MA/EP composite PCMs, and paraffin was chosen to encapsulate the composite PCMs avoiding liquid leakage. They also prepared the temperature control mortar and studied the physical mechanical properties. As can be seen in the open literature above, different kinds of composite PCMs by absorbing organic substances into EP were widespreadly prepared, but EP-based composite phase change building materials are a few studied [41,42,43,44] until now.

Cement board that is produced by mixing up Portland cement, EP and water has wide applications in buildings such as creating a smooth surface to walls and thermal insulation structure due to the EP’s higher porosity. In the current work, we focus on the incorporation of the RT28/EP composite PCM into ordinary cement to produce thermal energy storage cement board. A systematic experimental study to analyze the important effects of the thermo-mechanical properties of the produced samples, like apparent density and compression behaviors and thermal conductivity was performed. Moreover, the thermal energy storage performances of the cement boards containing different mass percentage of the RT28/EP composite PCM were evaluated. The goal of this study was to demonstrate the feasibility of using EP-based composite PCM in cement boards to increase their thermal inertia and to reduce the energy demand of the building.

## 2. Experimental Section

### 2.1. Preparation and Characterization of RT28/EP Composite Phase Change Materials (PCMs)

RT28, a kind of liquid saturated hydrocarbons, was purchased from Rubitherm GmbH corp. in Berlin, Germany. RT28 as phase change material can be applied in buildings due to its phase change temperature (about 28 °C) in the range of human comfort temperature (between 18 and 28 °C) [19]. EP was used as received. RT28/EP composite PCMs with different mass fractions of RT28 were prepared by adsorbing different amount of RT28 into the pores of EP at 60 °C for 1 h, respectively.

The microstructures of EP and the RT28/EP composite PCMs were observed using a scanning electron micro-scope (SEM, S-3700N, Hitachi, Kyoto, Japan), respectively. The thermal properties of RT28 and the RT28/EP composite PCMs were measured by using a differential scanning calorimeter (DSC, DSC2910, TA Instruments, New Castle, DE, USA) under N_2_ atmosphere. The measuring temperature ranged from −10 to 60 °C. The temperature rise rate was 5 °C/min.

### 2.2. Fabrication and Characterization of Cement Boards Containing RT28/EP Composites

Four kinds of slurries were prepared by mixing cement and water with the RT28/EP composite PCMs containing 10, 20, 30 and 40 wt % of RT28, respectively, in which all the mass ratios of the composites to cement to water are 1:1.5:2. The prepared slurries were formed into cubes by using a standard stainless steel mold (size: 70.7 mm × 70.7 mm × 70.7 mm) to measure their mechanical properties and into boards by using a home-made stainless steel mold (size: 100 mm × 100 mm × 10 mm) to measure their thermal conductivity and evaluate their thermal energy storage performance, respectively. Four kinds of the cubes and boards were obtained, in which the mass fractions of RT28 were calculated to be 4%, 8%, 12% and 16%, respectively. For comparison purpose, the EP powder was also mixed with cement and water at the same mass ratio to obtain the blank slurry followed by forming into the cubes and boards using the same fabrication process.

The mechanical properties of the cubes with different mass fractions of RT28 were evaluated by measuring their compressive strength and apparent density, respectively. After being kept under a moist atmosphere for about 24 h, all the fabricated cubes were maintained under water at 20 ± 1 °C for 7 days. Then, some of the cubes used for measuring the compressive strength were kept under a controlled condition (20 ± 2 °C in temperature and 60% ± 3% in humidity) until the tests were performed on a compression-testing machine (5000 A, Jinan Shijin Group Co., Ltd., Jinan, China). Other cubes used for measuring the apparent density were dried at 100 °C until their weight didn’t change. The accurate dimensions of the cubes were measured using a vernier caliper with a precision of 0.02 mm, and their weights were measured using an analytical balance with a precision of 0.001 g, respectively. The apparent density (ρ) of the cubes was calculated by the following formula: ρ= *m*/*V*, respectively, where *m* represents the weights of the cubes (unit: g), and *V* represents the volumes of the cubes (unit: cm^3^). Conforming to Chinese Standard JGJ/T 70-2009 [45], six same samples were prepared for each compressive strength and apparent density measurements, all measurements were arithmetically averaged.

The thermal conductivity of the fabricated boards was measured at room temperature using a hot disk thermal constant analyzer (Hot Disk TPS2500, Hot Disk AB Company, Uppsala, Sweden). The measurement accuracy of the thermal conductivity was within ±3%. The arithmetically average value of thermal conductivity for three times measurements was chose as the experimental result for each sample.

### 2.3. Thermal Energy Storage Performance Evaluation of Cement Boards Containing RT28/EP Composites

**Figure 1:**
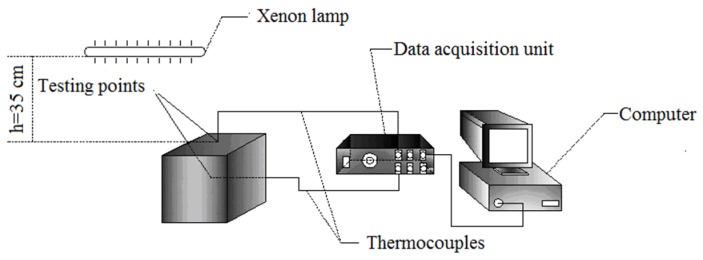
An experimental schematic drawing for testing energy conservation performance of composite boards.

A sketch of the experimental apparatus for testing the thermal energy storage performance of the composite boards (size: 100 mm × 100 mm × 10 mm) is shown in Figure 1. A small test room (100 mm × 100 mm × 100 mm) that consists of 6 pieces of the boards was set up below a halogen tungsten lamp (500 W) at a distance of 35 cm, which was used as the light source to simulate the sun. The radiation intensity was about 60 mw/cm^2^ measured by a radiometer (FZ-A type, Beijing Normal University) at the position. Two K-typed thermocouples linked to a data acquisition/switch unit (Agilent 34970A) were used to monitor the temperature variation of the test room. The thermocouples were calibrated before use, the accuracy was ±0.2 °C. One of them was placed at the outside surface of the top board, and the other one was placed in the center of the test room for recording the indoor temperature. When the lamp was switched on, the temperatures at the two spots of the test room started to be monitored. After 1.5 h, the lamp was switched off. The monitoring of the two temperatures continued until the test room cooled down to room temperature. The difference between the two temperatures varying with time was used to evaluate the performance of the boards with different mass fractions of RT28.

## 3. Results and Discussion

### 3.1. Characterization of RT28/EP Composite PCMs

In order to obtain form-stable RT28/EP composite PCMs, the maximum absorption ratio of RT28 in the EP powder should be determined. Firstly, the prepared RT28/EP composite PCMs with 40, 45 and 50 wt % of RT28 were placed on three piece of filter paper, respectively, followed by putting them into an oven at 60 °C for 30 min; then, the three pieces of filter paper were taken out from the oven and cooled down to room temperature; finally, the composite PCMs were removed from the three pieces of filter paper, respectively. We checked these pieces of filter paper carefully to make sure if there were any traces of RT28 left in them due to the leakage of RT28 from the pores of EP during the heating. It is shown that there is no trace of RT28 left in the filter paper on which the RT28/EP composite PCM containing 40 wt % of RT28 has been placed. However, as the mass fraction of RT28 is increased to 45%, the trace of RT28 is observed, implying that the maximum adsorption ratio of RT28 in EP is around 40%. Therefore, the RT28/EP composite PCMs containing no more than 40 wt % of RT28 can be considered as the form-stable composite PCMs.

**Figure 2:**
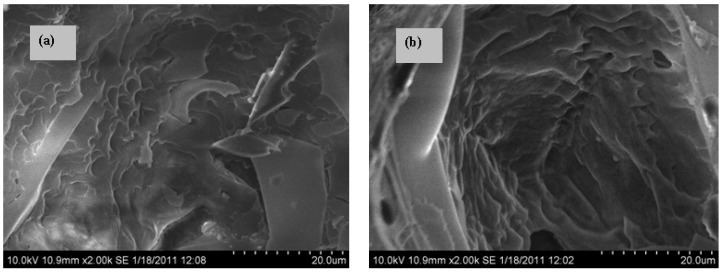
SEM images of the RT28/EP composite PCMs with 30 wt % (**a**) and 40 wt % (**b**) of RT28.

EP possesses a porous structure, which makes it possible to adsorb organic PCMs. Figure 2 shows the SEM images of the RT28/EP PCMs with 30 wt % (a) and 40 wt % (b) of RT28, respectively. We can see that the adsorbed RT28 is uniformly confined into the pores of EP, and the layer of RT28 becomes thicker as its mass fraction is increased from 30 to 40 wt %.

**Figure 3:**
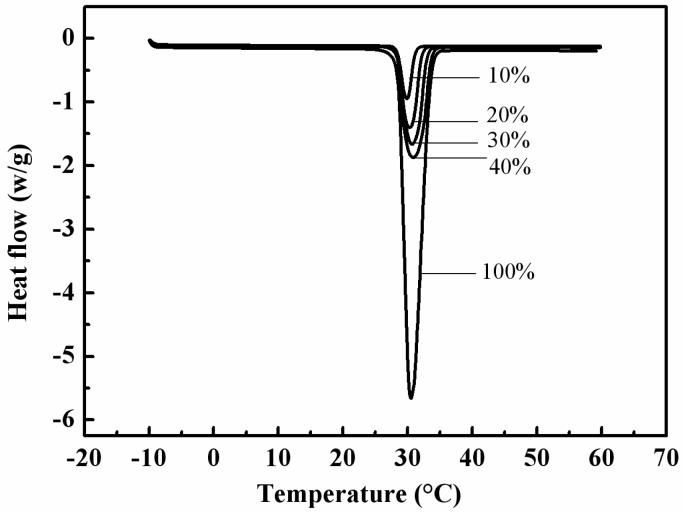
DSC curves of RT28 and the RT28/EP composite PCMs with 10, 20, 30 and 40 wt % of RT28, respectively.

Figure 3 displays the DSC curves of EP and the RT28/EP composite PCMs with 10, 20, 30 and 40 wt % of RT28, respectively. The melting temperatures were measured to be 28.41, 28.43, 28.40 and 28.48 °C for the composite PCMs containing 10, 20, 30 and 40 wt % of RT28, respectively, which are very close to 28.55 °C of RT28. RT28 has a melting latent heat as high as 197.8 kJ/kg. The melting latent heat values of the RT28/EP composite PCMs with 10%, 20%, 30% and 40% of RT28 were measured to be 18.11, 39.11, 58.14 and 77.44 kJ/kg, respectively. Obviously, the measured latent heat values of the composite PCMs are almost equivalent to their calculated latent heat values based on the mass fractions of RT28. It is revealed that the combination of RT28 with EP doesn’t change the phase change temperature of RT28, and the maximum latent heat value of the form-stable RT28/EP composite PCMs can reach as high as 77.44 kJ/kg.

### 3.2. Properties of Cement Cubes and Boards Containing RT28/EP Composite PCMs

**Figure 4:**
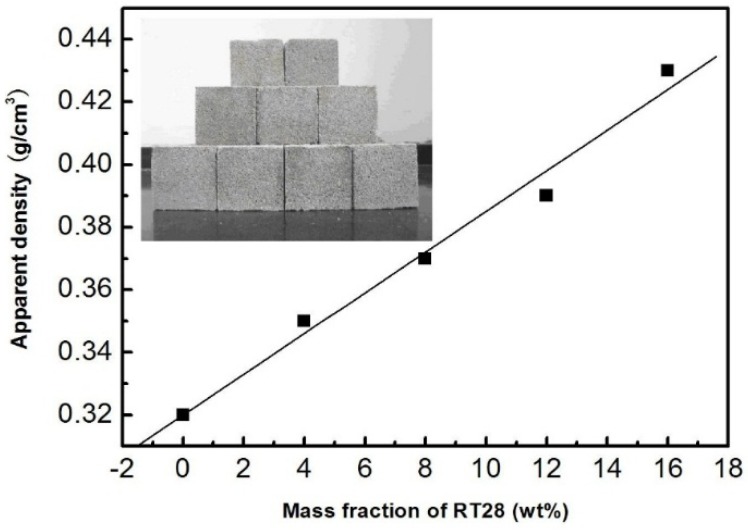
Relation between the apparent density of the cubes and the mass fraction of RT28 (the insert is the photograph of the cubes).

Figure 4 displays the apparent density of the four kinds of cubes made of cement and the RT28/EP composite PCMs with 10, 20, 30 and 40 wt % of RT28, respectively, together with that of the cube made of cement and EP. The photograph of the cubes is inserted in this figure. The mass fractions of RT28 in the five kinds of cubes were calculated to be 0%, 4%, 8%, 12% and 16%, respectively. The apparent density of the kind of cubes made of cement and EP was measured to 0.32 g/cm^3^ on average. For the composite cubes made of cement and the RT28/EP composite PCMs, their apparent density was measured to be 0.35, 0.37, 0.39 and 0.43 g/cm^3^ as the mass fraction of RT28 in the cubes was 4, 8, 12 and 16 wt %, respectively. The relationship between the apparent density (ρ) of the cubes and the mass fraction (*x*) of RT28 can be fitted into a linear equation: ρ = 0.32 + 0.0065*x*. It is revealed that the apparent density of the cubes increases linearly with the mass fraction of RT28. Since RT28 is adsorbed into the pores of EP, the volumes of the RT28/EP composites may not obviously increase with the mass fraction of RT28, but their weight definitely increases accordingly. Therefore, the apparent density of the composite cubes increases with the mass fraction of RT28. Note that the apparent density of the nine kinds of cubes is within the range from 0.3 to 0.8 g/cm^3^, indicating that all the cubes are up to standard.

The porosity of the composite cubes made of cement and the RT28/EP composite PCMs were estimated from the bulk density, the matrix density assuming that the cement pores are filled of air which remains trapped once the cement has solidified [46]. For the composite cubes made of cement and the RT28/EP composite PCMs, their porosity was calculated to be 0.383, 0.351, 0.339 and 0.313 as the mass fraction of RT28 in the cubes was 4, 8, 12 and 16 wt %, respectively.

**Figure 5:**
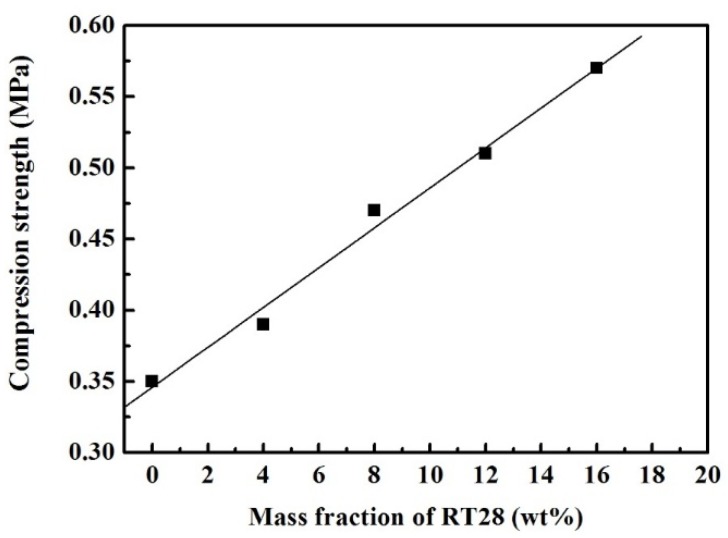
Relation between the compression strength of the cubes and the mass fraction of RT28.

Figure 5 shows the compressive strength of the five kinds of cubes containing 0%, 4%, 8%, 12% and 16% of RT28, respectively. The compression strength of the cube containing no RT28 is measured to be 0.35 MPa on average. As the mass fraction of RT28 in the cubes is increased to 4%, 8%, 12% and 16%, their compression strength accordingly increases to 0.39, 0.47, 0.51 and 0.57 MPa, respectively. The relationship between the compression strength (*S*) and the mass fraction (*x*) of RT28 can be also fitted into a linear equation: *S* = 0.346 + 0.014*x*. It is indicated that the compression strength of the cubes linearly increases with the mass fraction of RT28. Note that all the compression strength of the nine kinds of cubes is more than 0.3 MPa, indicating that all the cubes are up to standard. Obviously, the addition of RT28 not only endows the building materials with the thermal energy storage performance but also improves their mechanical properties.

**Figure 6:**
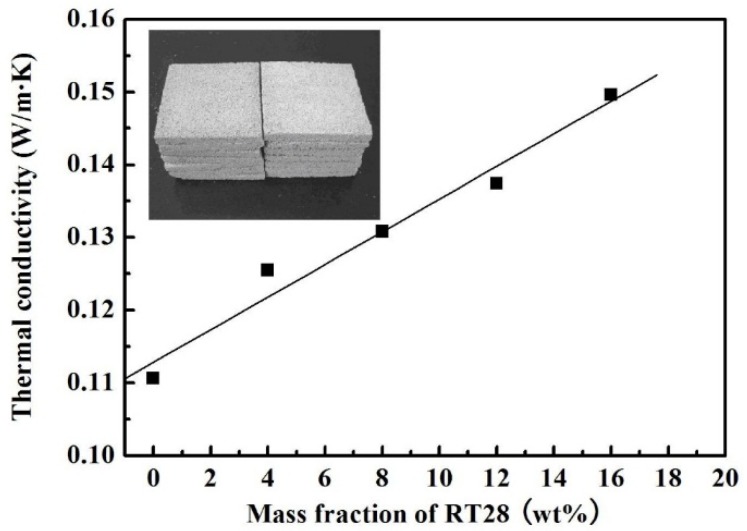
Relation between the thermal conductivity of the boards and the mass fraction of RT28 (the insert is the photograph of the boards).

Figure 6 shows the thermal conductivity of the five kinds of boards containing 0%, 4%, 8%, 12% and 16% of RT28, respectively. The photograph of the composite boards is inserted in this figure. The thermal conductivity of the board without RT28 is measured to be 0.11 W/m·K. As the mass fraction of RT28 in the boards is increased to 4%, 8%, 12% and 16%, their thermal conductivity increases to 0.126, 0.131, 0.137 and 0.149 W/m·K, respectively. The relationship between the thermal conductivity (*S*) and the mass fraction (*x*) of RT28 can be also fitted into a linear equation: *S* = 0.113 + 0.00225*x*. As the mass fraction of RT28 in the composite PCMs is increased, the pores of EP occupied by air are gradually filled with RT28. Since thermal conductivity of RT28 (0.276 W/m·K) is far larger than that of air (0.023 W/m·K), the thermal conductivity of the composite boards increases with the mass fraction of RT28.

### 3.3. Thermal Energy Storage Performance of Cement Boards Containing RT28/EP Composites PCMs

To explore the thermal energy storage performance of the five kinds of boards containing 0, 4, 8, 12 and 16 wt % of RT28, respectively, the test room was set up in two ways. In one way, the test room was set up using six identical boards, in which the mass fractions of RT28 were 0, 4, 8, 12 and 16 wt %, respectively. In the other way, except the top board, the other five boards of the test room contained no RT28. The boards with 0, 4, 8, 12 and 16 wt % of RT28 were used as the top board, respectively.

**Figure 7:**
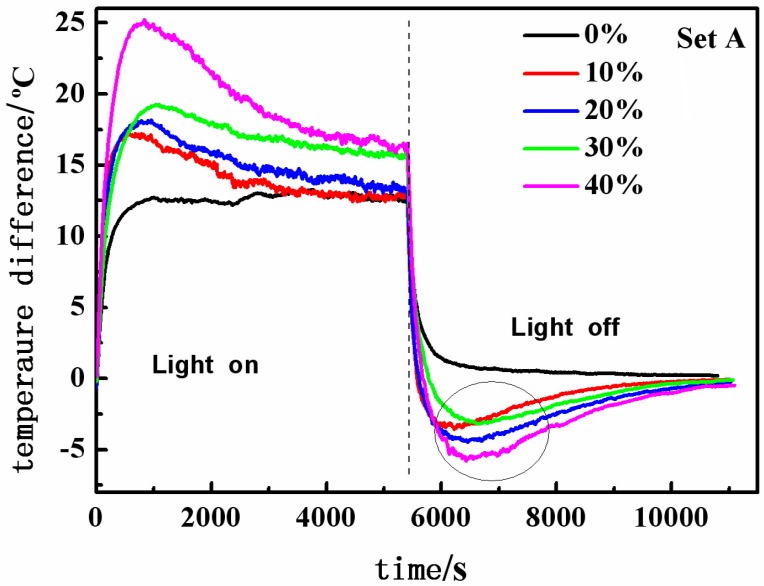
Variations of the temperature differences with time for the five test rooms that consist of six identical boards containing 0, 4, 8, 12 and 16 wt % of RT28, respectively (Set A).

Figure 7 shows the variations of the temperature differences with time for the test rooms set up using six identical boards containing 0, 4, 8, 12 and 16 wt % of RT28, respectively. The temperature difference is defined as the difference of temperature at outside surface of the top board from the indoor temperature of test room. For the test room with the boards containing no RT28, once the light source was switched on, the temperature at the outside surface of the top board increased quickly, resulting in a sharp rise in the temperature difference; 900 s after the light switching on, the temperature difference got stability around 13 °C; after the light was turned off, the temperature difference quickly dropped to zero. The temperature at the outside surface of the top board was always higher than the indoor temperature during the light switching on, and the maximum temperature difference was calculated to be 13.3 °C. It is revealed that the boards made of cement and EP exhibit the heat insulation capacity to prevent the rapid rise in the indoor temperature.

It can be also seen from Figure 7 that, the temperature differences in the test rooms that consist of six composite boards are larger than that in the test room with the boards containing no RT28, and the temperature differences increase with the mass fraction of RT28 in the boards. The maximum temperature differences are 17.2, 18.2, 19.3 and 25.2 °C for the test rooms set up using the composite boards containing 4, 8, 12 and 16 wt % of RT28, respectively, which are higher than 13.3 °C of the test room with the boards containing no RT28. The bigger the temperature difference, the lower the room temperature. It is revealed that, the incorporation of RT28 into the boards can improve their heat insulation capacity, and the performance enhances with the increase in the mass fraction of RT28. During the illumination of the light source, RT28 integrated into the boards took the phase change from solid state to liquid state by absorbing some amount of heat coming from the light source. Since some heat has been absorbed by RT28, the indoor temperatures of the test rooms set up using the composite boards should be lower than that of the test room set up using the boards without RT28. The amount of the heat absorbed by RT28 is increased with the mass fraction of RT28 in the boards, resulting in the rise in the temperature difference accordingly. More significantly, as shown in Figure 7, during the cooling, for every test room that consisted of the composite boards, the temperature at the outside surface of the top board is even lower than the indoor temperature of the test room (as shown with a circle in Figure 7). It is revealed that the composite boards containing RT28 have the function of keeping the test room warm during the cooling, whereas the boards containing no RT28 don’t possess the function. The function enhances with the increase in the mass fraction of RT28 in the boards. After the light was switched off, RT28 integrated into the boards took the phase change from liquid state to solid state, and simultaneously released some amount of latent heat. It is reasonable that the indoor temperatures of the test rooms that consist of the composite boards are higher than that of the test room with the boards containing no RT28, owing to the latent heat released by RT28. Since the amount of heat released by RT28 is increased with the mass fraction of RT28 in the boards, the function of keeping the rooms warm enhances accordingly.

**Figure 8:**
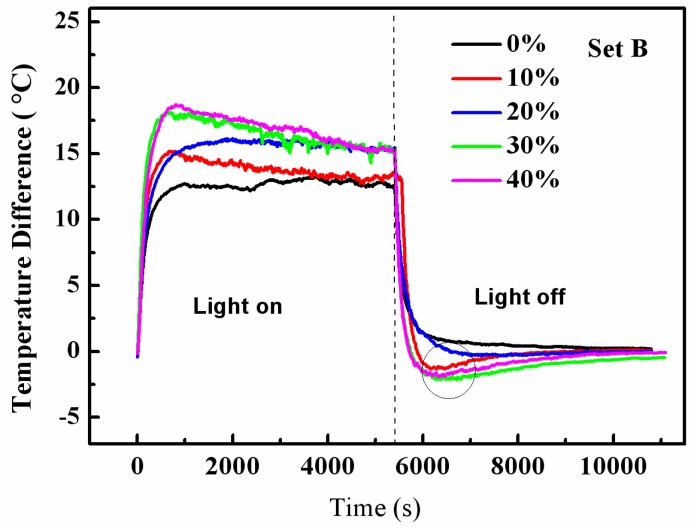
Variations of the temperature differences with time for the five test rooms composed of the top boards containing 0, 4, 8, 12 and 16 wt % of RT28, respectively (Set B).

Figure 8 shows the variations of the temperature differences with time for the test rooms with the top boards containing 0, 4, 8, 12 and 16 wt % of RT28, respectively. It can be seen that the curves in Figure 8 have similar trends to those in Figure 7. During the illumination, the maximum temperature differences for the test rooms with the top boards containing 4, 8, 12 and 16 wt % of RT28 are 15.2, 16.2, 18.2 and 18.7 °C, respectively, higher than 13.3 °C of the test room with the boards containing no RT28. It is suggested that the integration of RT28 into the top board of the test room can also exhibit the increase in thermal mass, and the performance gradually enhances with the increase of the mass fraction of RT28. During the cooling, the temperatures at the outside surfaces of the top boards are even lower than the indoor temperatures, indicating that the top boards containing RT28 also exhibit the function of keeping the rooms warm. It is reasonable that, at the same mass fraction of RT28, the test rooms composed of the six composite boards show superior heat insulation capacity to the test room with only the top board containing RT28. The more amount of the PCM is integrated into a building, the better energy conservation performance can be reached.

## 4. Conclusions

Form-stable RT28/EP composite PCMs can be obtained by limiting the mass fraction of RT28 no more than 40 wt %. The adsorbed RT28 is observed to be uniformly confined into the pores of EP. The phase change temperatures of the RT28/EP composite PCMs with different mass fractions of RT28 are very close to that of the pure RT28. The latent heat values of the composite PCMs are almost equivalent to the calculated latent heat values based on the mass fractions of RT28. The apparent density and compression strength of the composite cubes increase linearly with the mass fraction of RT28. The thermal conductivity of the composite boards increases with the mass fraction of RT28. The composite boards containing RT28 not only exhibit the increase in thermal inertia during the illumination of a light source but also have the function of keeping the test rooms warm after the light source is turned off, which makes the RT/EP composite PCMs show great promise for improving the human comfort and reducing the energy consumption in the buildings.
